# Knowledge, attitudes, and practices regarding antimicrobial use and antimicrobial resistance among medical students and house surgeons in India: a call to reform medical curricula and training practices

**DOI:** 10.3389/fpubh.2026.1876665

**Published:** 2026-07-31

**Authors:** Shaheer Aboobacker, Ijyaa Singh, Gauri E. Yadav, Hyfa Mohammed Ali, Sudhi Nath, Sandhya S. Kulkarni, G. K. Mini, S. S. Lal

**Affiliations:** 1 ReAct Asia Pacific, Thiruvananthapuram, India; 2 International Health Policy Program Foundation, Nonthaburi, Thailand; 3 MIMER Medical College, Talegaon Dabhade, Pune, Maharashtra, India; 4 Global Institute of Public Health, Ananthapuri Hospitals and Research Institute, Thiruvananthapuram, India

**Keywords:** antibiotic use, antimicrobial resistance, antimicrobial stewardship, cross-sectional study, India, knowledge, attitude and practice, medical curriculum

## Abstract

**Background:**

Antimicrobial resistance (AMR) is a major global public health threat driven by the overuse and misuse of antimicrobials across sectors. Medical students, as future prescribers, play a crucial role in combating AMR. This study aimed to assess the knowledge, attitudes, and practices (KAP) regarding antimicrobial use and AMR among medical students and house surgeons in India.

**Methods:**

A cross-sectional survey was conducted among medical students and house surgeons in Maharashtra, India, using a previously validated questionnaire administered via Google Forms. Data were analyzed using R statistical software. Descriptive statistics were used, and inferential analyses included chi-square tests, Kruskal–Wallis tests, Dunn's *post hoc* tests, and Spearman correlation.

**Results:**

A total of 414 participants were included. The overall knowledge score was 69%, indicating moderate knowledge. While 90.1% recognized that AMR affects all age groups, only 52.7% were aware that resistant bacteria can spread between individuals, and 33.6% correctly understood that AMR results from microorganisms becoming resistant rather than the human body. Knowledge varied significantly across academic years (*p* < 0.001). More than 75% of participants demonstrated a positive attitude toward AMR as a major health threat; however, only 20.8% had received formal training on AMR. Regarding practices, 80.7% reported completing antibiotic courses, while 60.4% reported that they would avoid self-medication. Spearman correlation showed a positive association between knowledge and attitude (r = 0.39, *p* < 0.001) and weak negative correlations between knowledge and practice (r = −0.11, *p* = 0.03) and between attitude and practice (r = −0.10, *p* = 0.03).

**Conclusions:**

The study highlights moderate knowledge, positive attitudes, and suboptimal practices regarding AMR among medical students. Significant gaps in formal training and understanding persist. Early integration of AMR education into medical curricula, along with training in rational prescribing, patient assessment, and antimicrobial stewardship, is essential to curb the development and spread of AMR.

## Introduction

Antimicrobials, once a medical wonder used to treat and prevent infections in humans and animals, are now losing effectiveness due to the emergence of resistance. This has become one of the leading public health threats globally. Antimicrobial resistance (AMR) is estimated to have caused 4.95 million deaths in 2019, with 1.27 million directly attributed to bacterial AMR (1). The main cause of AMR is the misuse, overuse, and over-the-counter availability of antimicrobials contributing to emergence of resistance causing high fatalities. Approximately 5 million deaths annually are associated with bacterial infections involving AMR (excluding Mycobacterium tuberculosis), with 4.3 millions of these occurring in low- and middle-income countries (LMICs). If no urgent actions are taken, the number of deaths could rise to 10 million per year by 2050 (2).

AMR is not a concern just for the health care sector, it also affects other sectors like livestock and agriculture, where they are widely being used. The economic impact of it could be a loss of around US$100 trillion to the global economy by 2050 (2). A World Bank report states that AMR would decrease global exports by 3.8%, reduce livestock production by 7.5% per year, and increase healthcare-related costs of US$1 trillion by 2050 (3). This states the urgency and need for prioritization of AMR as a global emergency and for countries and organizations to develop policies and build systems to combat this growing menace.

The United Nations has prioritized AMR by issuing a political declaration in 2016 and reiterating every year on how much AMR has affected humanity and the ecosystem. Accordingly, two Sustainable Development Goals (SDG) indicators have been added specifically for AMR, which monitor the proportion of bloodstream infections due to *Escherichia coli* resistant to third-generation cephalosporins and methicillin-resistant *Staphylococcus aureus* (MRSA) (4). Other monitoring includes the Tracking AMR Country Self-Assessment Survey (TrACSS), which is done jointly by the World Health Organization (WHO), World Organization for Animal Health (WOAH), and Food and Agriculture Organization (FAO). These shall help understand not just the burden of AMR, its effects in the world, and the actions taken by countries against AMR.

It is predicted that 70% of AMR is in Asia, with Bangladesh, Nepal, and Sri Lanka having high levels of resistance to beta-lactams (5–8). In India, resistance to third and fourth generation cephalosporins among *E. coli* is 77% and 63% respectively (9). Children in Myanmar have shown high rates of carbapenem resistance for *E. coli, K. pneumoniae* and *Acinetobacter* sp. (10). Pakistan ranks third in antimicrobial consumption among LMICs (11) with a projected 44% increase in antimicrobial usage in food-producing animals from 2020–2030 (12).

There is an increasing amount of inappropriate antibiotic use, as ascertained from a study in China, which identified that of the patients assessed, only 8.7% of antibiotic prescriptions were appropriate (13). A study among physicians at the Royal Hospital, Oman, showed many factors associated with AMR, including inadequate handwashing, widespread use of antibiotics, prescribing broad-spectrum antibiotics, and inappropriate choice and duration of antibiotic therapy (14). A systematic review of the Knowledge, Attitude and Practices (KAP) on AMR in India showed that though healthcare workers have significant knowledge on AMR, there was a gap in its practice (15). A study among faculty, residents, and interns at a tertiary care setting in India also suggests a similar gap between knowledge and practice (16).

Medical students are the future generation of professionals responsible for diagnosing and prescribing treatments. It is of paramount importance that they have thorough knowledge of antimicrobials, rational antibiotic prescription practices, and AMR. They should be well informed of national and regional guidelines along with appropriate protocols and procedures for the responsible use of antimicrobials.

The objectives of the present study were to assess the level of knowledge and awareness among medical students regarding antimicrobial use and AMR in India and to identify gaps in their AMR-related knowledge and training exposure.

## Methodology

### Study design and setting

A cross-sectional survey was conducted to assess the knowledge, attitudes, and practices on AMR among medical students and house surgeons at a private Medical College and Hospital, from Western Maharashtra, India. The curriculum at the college follows a Competency Based Medical Education (CBME) prescribed by the National Medical Commission (NMC).

### Study population

The study population comprised MBBS (Bachelor of Medicine and Bachelor of Surgery) students and house surgeons enrolled at the institution in April 2024 (total population, *N* = 750). The sample size was calculated using a 50% population proportion, a 5% margin of error, and a 95% confidence interval. After applying finite population correction, the final sample size was determined to be 255. A convenience sampling method was employed, wherein all eligible students present during scheduled academic sessions were invited to participate. Each year, 150 students enroll in the college and of the 750 eligible students, 430 were approached during these sessions, of whom 414 completed the questionnaire, yielding a response rate of 96.3%.

### Inclusion and exclusion criteria

The participants were required to be registered and enrolled at the institution at the time of the study, either as MBBS students or as house surgeons. Students who were absent during the scheduled data collection sessions were not included, as participation was facilitated through in-person academic sessions.

### Data collection and tools

The data collection was done using a structured, pre-validated questionnaire adapted from a previously validated AMR KAP survey tool used in China (17). The adapted questionnaire was reviewed by a panel of subject matter experts including public health professionals and medical educators to ensure its relevance, clarity, and applicability in the Indian healthcare context. The questionnaire contained 25 questions to assess the knowledge, attitudes, and practices on AMR. The first part of the questionnaire dealt with demographic information like age, gender, and the year of study. The second part had ten questions on knowledge, which included Multiple-choice and True/False questions assessing factual understanding of AMR. These were coded as 0 for False and 1 for True. The third part also had twelve questions to assess Attitude with two being “yes” and “no” questions and the remaining were scored on a five-point Likert scale (Strongly Agree = 5 and Strongly Disagree = 1) with two Questions (9 and 10) that were reverse-coded to prevent response bias. The final part of the questionnaire assessed AMR Practices and had five questions, which included three True/False questions, one multiple-choice question, and one self-reported AMR training experience question.

The survey was conducted electronically via Google Forms, with QR codes generated for easy access. Participants were invited during scheduled academic sessions, and informed consent was obtained before participation by providing them a short summary of the study and that they have the choice of participating in the study by either choosing Yes or No. Confidentiality of data was maintained at all levels of the study by not revealing the participant's details anywhere.

### Grading methods

The questions of True/False type were given a score of 1 for the right answer and 0 for the wrong answer. Bloom's cutoff (18) was used to categorize knowledge scores as high (80%−100%), moderate (60–79%), and low (< 60%). Questions of a five-point Likert scale type were scored from 1 to 5 for strongly disagree, disagree, neutral, agree, and strongly agree, respectively.

### Data management and analysis

The data was downloaded from the Google form into an Excel sheet. The data was checked for correctness and completeness before analysis. All collected data were exported to R software (Version R 4.4.2) for statistical analysis. Descriptive statistics were used to summarize demographic characteristics and KAP responses. Statistical tests done to infer results were by using the chi-square test (to assess associations between categorical variables), Kruskal-Wallis (to compare knowledge scores across different academic years), and Dunn tests (for comparisons between academic year groups).

### Ethical approval

The study was approved by the ethical committee at Ananthapuri Hospitals and Research Institute, Thiruvananthapuram. The requisite documents were shared with the concerned institute, and permission from the head of the institute was taken before conducting the study.

### Consent to participate

The structured questionnaire was merged into an online Google form. The URL was distributed among the participants. A clear statement was included at the beginning of the questionnaire form that completion of it indicates the informed, voluntary consent of the participant to participate in the study by either marking yes or no.

## Results

The present study assessed the knowledge, attitude, and practice of MBBS students and house surgeons. A total of 414 medical students, including house surgeons, participated in the study. The study had 199 female and 215 male participants, with most of them in the age group 18–23 years (90%), while the rest were 24 years and above (10%) (Table 1).

**Table 1:** Demographic characteristics of participants (*N* = 414).

Variable	Category	*N* (%)
Gender	Female	199 (48.1)
	Male	215 (51.9)
Age group (years)	18–20	107 (25.8)
	21–23	266 (64.2)
	24 and above	41 (10)
Academic year	First MBBS	48 (11.6)
	Second MBBS	117 (28.3)
	Third MBBS	65 (15.7)
	Fourth year	96 (23.2)
	House surgeons	88 (21.2)

### Knowledge

Table 2 shows the number of participants who provided the correct answers to the Knowledge section questions in the survey. The responses showed that overall awareness of AMR was moderate. Of the participants, 88.9% responded that AMR is an issue not just in other countries but also in India and that it can affect people of all age groups (90.1%), only 52.7% agreed that resistant bacteria can spread from one person to another. Among the participants, 66.4% believe that AMR occurs when your body becomes resistant to antimicrobials and they no longer work as well, which is in fact, a false statement. 40% of the participants do not think that AMR could make surgeries, transplantations, and treatments more dangerous, and 70% understand that many organisms are becoming resistant to antibiotics, which will cause difficulty in treating them.

**Table 2:** Knowledge about AMR in medical students.

Question	1st Year (*n* = 48)	2nd Year (*n* = 117)	3rd Year (*n* = 65)	4th Year (*n* = 96)	House surgeons (*n* = 88)	Total (*n* = 414)
1. Antimicrobial resistance occurs when your body becomes resistant to antimicrobials, and they no longer work as well.	12 (25%)	46 (39.3%)	23 (35.4%)	27 (28.1%)	31 (35.2%)	139 (33.6%)
2. Many infections are becoming increasingly resistant to treatment by antimicrobials.	34 (70.8%)	89 (76.1%)	37 (56.9%)	72 (75%)	72 (81.8%)	304 (73.4%)
3. If bacteria are resistant to antimicrobials, it can be very difficult or impossible to treat the infections they cause.	33 (68.8%)	90 (76.9%)	34 (52.3%)	67 (69.8%)	74 (84.1%)	298 (71.9%)
4. Antimicrobial resistance is an issue in other countries but not here in India.	34 (70.8%)	110 (94%)	59 (90.8%)	89 (92.7%)	76 (86.4%)	368 (88.9%)
5. Antimicrobial resistance is only a problem for people who take antimicrobials regularly.	27 (56.2%)	88 (75.2%)	52 (80%)	81 (84.4%)	68 (77.3%)	316 (76.3%)
6. Antimicrobial resistant infections could make medical procedures like surgery, organ transplants and cancer treatment much more dangerous.	29 (60.4%)	63 (53.8%)	29 (44.6%)	59 (61.5%)	65 (73.9%)	245 (59.2%)
7. Bacteria which are resistant to antimicrobials can be spread from person to person.	23 (47.9%)	51 (43.6%)	25 (38.5%)	57 (59.4%)	62 (70.5%)	218 (52.7%)
8. AMR can affect only older population; children and youth are immune to it.	37 (77.1%)	104 (88.9%)	60 (92.3%)	89 (92.7%)	83 (94.3%)	373 (90.1%)
9. The overuse of antibiotics to treat, prevent and control infections in humans, animals and plants do not have any bearing on the emergence and spread of AMR.	32 (66.7%)	87 (74.4%)	58 (89.2%)	73 (76%)	62 (70.5%)	312 (75.4%)
10. Resistance to one specific antibiotic agent does not lead to the resistance to a whole related class.	25 (52.1%)	90 (76.9%)	52 (80%)	69 (71.9%)	58 (65.9%)	294 (71%)

**Figure 1:**
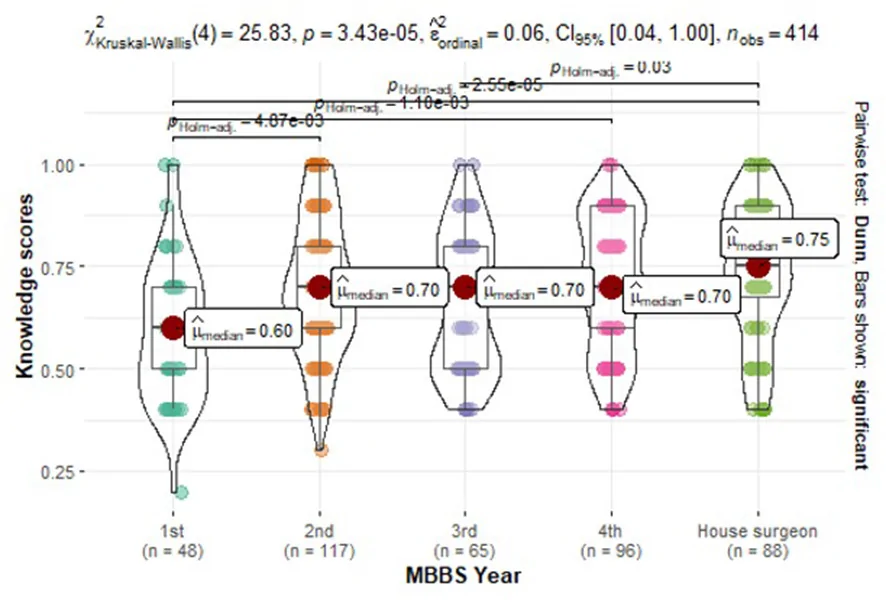
Kruskal-Wallis test between knowledge scores and the MBBS students according to the year.

A Kruskal-Wallis test done to compare knowledge score across different academic years showed a statistical significance (*p* = < 0.001). A *post hoc* Dunn test showed that there is significant difference in knowledge among the participants (Figure 1). As shown in the figure, the difference in knowledge is between first year students and third, fourth and house surgeons; and between third year students and house surgeons.

### Attitude

Attitude indicates the overall outlook and understanding toward antimicrobials and AMR. Most of the participants have given a Likert score of 4 and above. For the questions related to attitude, more than 75% of the participants gave a Likert score of 4 and above (Table 3). The overall attitude awareness toward AMR is high with 92.8% agreeing that antimicrobials are being misused and 87.4% think that antimicrobials are widely used in agriculture and food production in the country.

**Table 3:** Response to questions on attitude toward AMR.

Questions	Strongly agree (5)	Agree (4)	Neutral (3)	Disagree (2)	Strongly disagree (1)
1. Antimicrobial resistance is one of the biggest problems the world faces	45.9%	41.5%	10.9%	1.2%	0.5%
2. Everyone needs to take responsibility for using antimicrobials responsibly.	55.5%	35.5%	7.7%	0.9%	0.2%
3. I am worried about the impact that antimicrobial resistance will have on my health, and that of my family.	47.3%	38.6%	11.6%	1.4%	0.9%
4. Doctors should only prescribe antimicrobials when they are needed	59.2%	20.4%	8.4%	1%	1%
5. People should use antimicrobials only when they are prescribed by a doctor.	62.1%	28.5%	8.2%	1%	0.2%
6. Parents should make sure all of their children's vaccinations are up to date.	71.7%	19.8%	7.7%	0.7%	0%
7. Farmers should give fewer antimicrobials to food-producing animals	38.6%	39.6%	18.3%	2.2%	1.2%
8. People should wash their hands regularly	70.5%	21.7%	7%	0.7%	0%
9. There is not much people like me can do to stop antimicrobial resistance.	18.3%	25.4%	25.4%	21.7%	9.2%
10. I am not at risk of getting an antimicrobial resistant infection, as long as I take my antimicrobials correctly.	20.5%	31.9%	25.6%	15.7%	6.3%

Number in brackets indicate Likert scale codes. Reverse coding for question 9 and 10.

### Practice

More than 80% of participants supported the statement that once treatment commences, one should stop antimicrobials only after the completion of the course (Table 4). More than 70% stated that they wouldn't ask doctors to prescribe antimicrobials for a common cold and will not take the same medications as their family or friends who had taken antimicrobials for similar illness. Only 60.4% reported that they would refrain from self-medication if they experienced similar symptoms in the future.

**Table 4:** Self-reported practices on when to stop antimicrobial therapy.

Options	1st Year (*n* = 48)	2nd Year (*n* = 117)	3rd Year (*n* = 65)	4th Year (*n* = 96)	House surgeons (*n* = 88)	Total (*n* = 414)
1. When you've taken all of the antimicrobials as directed	68.7%	74.4%	73.8%	88.5%	92.1%	80.7%
2. When you feel better	25%	13.7%	18.5%	7.3%	5.7%	12.6%
3.When you run out of antimicrobials	2.1%	3.4%	1.5%	1.1%	1.1%	1.9%
4. I don't know	4.2%	8.5%	6.2%	3.1%	1.1%	4.8%

**Figure 2:**
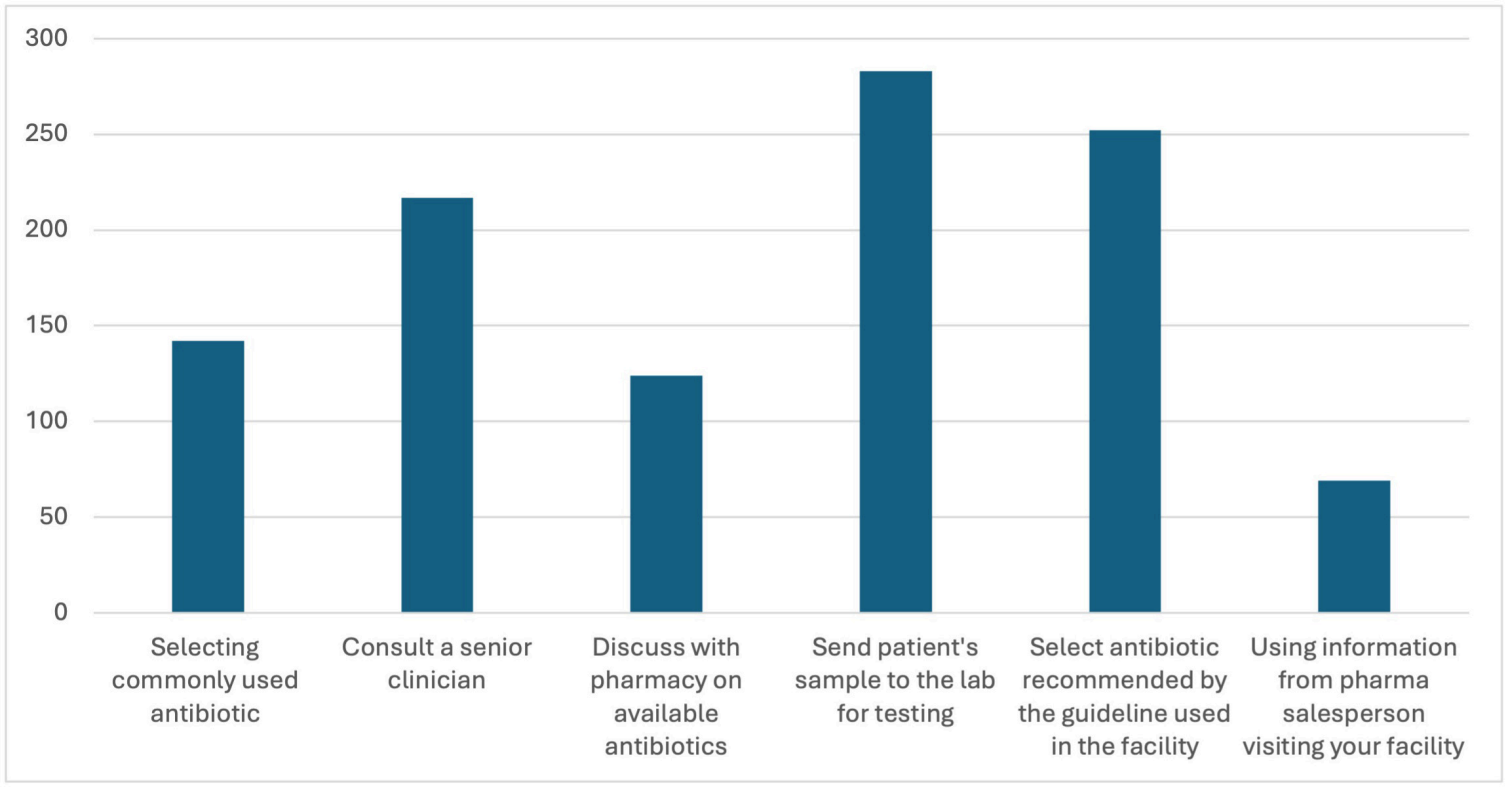
Decision making practices for antibiotic selection in clinical settings.

Figure 2 shows the response of the participants on the usage of antibiotics for infections. More than 60% of the participants said that they would conduct laboratory investigations (68.3%) for patients and use standard treatment guidelines (60.9%) for treatment. Over 50% of the participants opted to consult a senior clinician while 34.3% and 29.9% said that they would select the antibiotics used in the respective departments and discuss with the pharmacy, using the available antibiotics respectively. Among the participants, 16.7% said that they would prescribe antibiotics based on the information provided by the pharmaceutical salesperson visiting the hospital.

**Figure 3:**
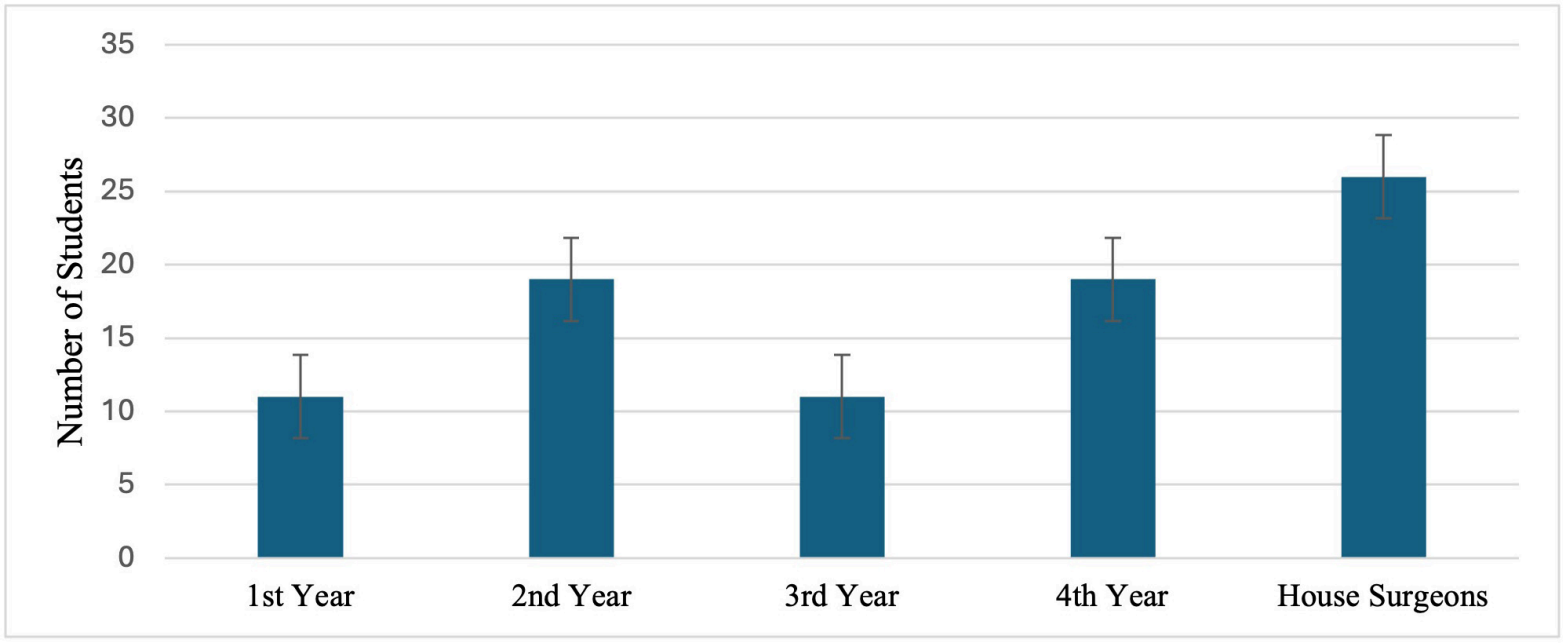
AMR training received during MBBS course- students' subjective perception.

Based on student's subjective self-reported experience, only 86 (20.8%) participants received any training on AMR while 328 (79.2%) received no training. Training received during the course of study is displayed in Figure 3.

Spearman correlation showed a positive and significant correlation between knowledge-attitude (0.39, *p* value < 0.001), negative and significant correlations between knowledge-practice (−0.11, *p* value = 0.03) and attitude-practice (−0.1, *p* value = 0.03) (Table 5).

**Table 5:** Spearman correlation between scores of knowledge, attitude and practices.

Variable	Knowledge	Attitude	Practice
Knowledge	1	-	-
Attitude	0.39 (< 0.001)	1	-
Practice	−0.11 (0.03)	−0.1 (0.03)	1

## Discussion

This study explored the knowledge, attitude and practices of medical students of all phases of the medical course toward antimicrobial use and AMR. As students in healthcare, it is imperative that they have thorough knowledge and understanding of proper antimicrobials usage and the detrimental health impacts of AMR. They are also the gateway of health-related information for friends and families who rely on them for medical guidance.

Though most of the participants agreed that AMR is a global concern and that it can affect people of all ages, there were notable gaps in the basic knowledge about AMR among the participants. This is similar to a review done earlier regarding knowledge of medical students where, though AMR was recognized as a major problem, the students lacked basic knowledge regarding AMR (19). The overall Knowledge level was moderate, with a score of 69% based on Bloom's criteria (18). The general understanding that AMR occurs when your body becomes resistant to antimicrobials (66.4%), whereas it is the microbes that become resistant to the drugs, is concerning. Another finding that 40% do not think that AMR could make surgeries, transplantations and treatments more dangerous is important. In addition, 47.3% of the participants don't understand that resistant bacteria can spread from one person to another. Therefore, there is an issue with the basic understanding of AMR. However, the knowledge gap reduces as the participants are from a higher year of study, which could be due to them learning more about drugs, prescription practices and interacting with patients. There is a need to bridge this gap by incorporating AMR into the curricula specifically providing them with better understanding of AWaRe classification of drugs, rational empirical use of antibiotics and utilizing diagnostics optimally before and after starting antimicrobial therapies for infections.

A positive attitude was evident in understanding AMR in the present study which is necessary and important to implement in everyday practice. The participants agreed that antimicrobials are being misused (92.8%) and are being widely used in agriculture and food production in the country (87.4%). This shows that their attitudes are aligned to a one-health approach which can contribute to efforts toward resisting AMR (20). The participants understand that AMR is a major threat the world faces and there needs to be judicious utilization of antimicrobials in all sectors, be it human, animals and plants. COVID-19 has taught us how important Infection, Prevention and Control (IPC) practices play in preventing infections and the present study reiterates this fact (21).

Good practices with regards to antibiotics include adhering to proper guidelines, completing the prescribed course, not taking unwanted antimicrobials and promoting such practices. There is a good understanding on antimicrobial course completion, the harm of self-medications and non-requirement of antimicrobials for viral infections. 24.6% mentioned using leftover medicines, which is less compared to 28.7% from a similar study in India (22).

A major component missing is the training on AMR which needs to be addressed. A considerable revision of the medical curricula and training should be done, factoring in early integration of AMR into medical education. The future practitioners and public health professionals who are going to guide the community and health systems should have adequate knowledge of AMR. If they don't realize the importance of judicious antimicrobial prescription, the catastrophe it shall lead to cannot be controlled or prevented.

Medical curriculum assessment must be done using a standardized tool like the AMR Curriculum Assessment Tool for Medical Education developed by WHO (23). It will help in evaluating the AMR content in the curriculum and strategizing training modules for enhancing AMR knowledge and understanding of medical students. Apart from reforming this, there need to be programs, specific trainings, stewardship courses, seminars and campaigns regarding AMR as suggested in similar studies from other countries (24–27).

## Limitations

This study has a few limitations worth noting. The single-institution design restricts the generalizability of findings to the broader Indian medical student population. Additionally, the cross-sectional design precludes any causal inference between knowledge, attitude, and practice with a qualitative study or mixed-methods study providing further insights. There is also the chance for response bias during the completion of questionnaire. As data were collected through self-reported questionnaires, the possibility of social desirability and self-reporting bias cannot be entirely ruled out. Also, AMR training was assessed using a single self-reported item and as such the questionnaire did not capture the content of the training, which precludes any characterization of its structure or quality. The study being a KAP study couldn't assess the curriculum as a whole and a specific study evaluating the curriculum is necessary to identify gaps and provide the apt recommendations based on it.

## Conclusion

This study highlights the gaps in knowledge about AMR among medical students and house surgeons trained under the standardized NMC-approved curriculum, which is uniformly implemented across all medical colleges in India. The findings reveal moderate knowledge, positive attitudes, and suboptimal practices regarding antimicrobial use and resistance, with a significant proportion of participants lacking formal AMR training. Since the curriculum is common to all medical institutions in India, these gaps likely reflect a systemic issue rather than an institution-specific one, strengthening the case for policy reformation at the national level. Training in judicious use of antimicrobials, patient assessment, and antimicrobial stewardship should be prioritized within the NMC framework, ensuring future physicians across India are equipped to address AMR challenges and promote rational antimicrobial use.

## Data Availability

The raw data supporting the conclusions of this article will be made available by the authors, without undue reservation.

## References

[1] Antimicrobial Resistance Collaborators. Global burden of bacterial antimicrobial resistance in 2019: a systematic analysis. Lancet (2022) 399:629–55. doi: 10.1016/S0140-6736(21)02724-0 35065702 PMC8841637

[2] O'Neill J. Tackling Drug-Resistant Infections Globally: an Overview of Our Work. Review on Antimicrobial Resistance. (2016).

[3] By 2050 Drug-Resistant Drug-Resistant Infections Could Cause Global Economic Damage on Par with 2008 Financial Crisis. World Bank. n.d. Available online at: https://www.worldbank.org/en/news/press-release/2016/09/18/by-2050-drug-resistant-infections-could-cause-global-economic-damage-on-par-with-2008-financial-crisis (Accessed April 7, 2026).

[4] Global Indicator Framework for the Sustainable Development Goals and Targets of the 2030 Agenda for Sustainable Development. Available online at: https://unstats.un.org/sdgs/indicators/Global-Indicator-Framework-after-2024-refinement-English.pdf (Accessed April 7, 2026).

[5] Kang C-I Song J-H. Antimicrobial resistance in Asia: current epidemiology and clinical implications. Infect Chemother. (2013) 45:22–31. doi: 10.3947/ic.2013.45.1.22 24265947 PMC3780932

[6] Ahmed I Rabbi MB Sultana S. Antibiotic resistance in Bangladesh: a systematic review. Int J Infect Dis. (2019) 80:54–61. doi: 10.1016/j.ijid.2018.12.017 30634043

[7] Ariyawansa S Gunawardana KN Hapudeniya MM Manelgamage NJ Karunarathne CR Madalagama RP . One health surveillance of antimicrobial use and resistance: challenges and successes of implementing surveillance programs in Sri Lanka. Antibiotics. (2023) 12:446. doi: 10.3390/antibiotics12030446 36978313 PMC10044479

[8] Kakchapati S Rijal A Kc SP. Antimicrobial resistance in Nepal: the next invisible pandemic. Health Prospect. (2021) 20:22–4. doi: 10.3126/hprospect.v20i1.41210

[9] National Antimicrobial Resistance Surveillance Network Annual Report 2021. Available online at: https://ncdc.mohfw.gov.in/uploads/pdf/amr36.pdf (Accessed April 7, 2026).

[10] San T Aung MS San N Aung MMZ Mon WLY Thazin TE . Bacterial species and antimicrobial resistance of clinical isolates from pediatric patients in Yangon, Myanmar, 2020. Infect Dis Rep. (2022) 14:26–32. doi: 10.3390/idr14010004 35076535 PMC8788269

[11] Majid Aziz M Haider F Rasool MF Hashmi FK Bahsir S Li P . Dispensing of non-prescribed antibiotics from community pharmacies of Pakistan: a cross-sectional survey of pharmacy staff's opinion. Antibiotics. (2021) 10:482. doi: 10.3390/antibiotics10050482 33922058 PMC8143445

[12] Mulchandani R Wang Y Gilbert M Van Boeckel TP. Global trends in antimicrobial use in food-producing animals: 2020 to 2030. PLoS Glob Public Health. (2023) 3:e0001305. doi: 10.1371/journal.pgph.0001305 36963007 PMC10021213

[13] Chang Y Chusri S Sangthong R McNeil E Hu J Du W . Clinical pattern of antibiotic overuse and misuse in primary healthcare hospitals in the southwest of China. PLoS ONE. (2019) 14:e0214779. doi: 10.1371/journal.pone.0214779 31242185 PMC6594576

[14] Rahbi FA Salmi IA Khamis F Balushi ZA Pandak N Petersen E . Physicians' attitudes, knowledge, and practices regarding antibiotic prescriptions. J Glob Antimicrob Resist. (2023) 32:58–65. doi: 10.1016/j.jgar.2022.12.005 36584969

[15] Rana S Kaur KN Narad P Walia K Saeed S Chandra A . Knowledge, attitudes and practices of antimicrobial resistance awareness among healthcare workers in India: a systematic review. Front Public Health. (2024) 12:1433430. doi: 10.3389/fpubh.2024.1433430 39104891 PMC11298436

[16] Balaji L V K A Nandhagopal M Subramaniam J. Antimicrobial archetypes: assessing the Knowledge, Attitude, and Practice (KAP) trends in Antimicrobial Resistance (AMR) and Antimicrobial Stewardship Program (AMSP) among faculties, residents, and interns in a tertiary care hospital. Cureus. (2024) 16:e64722. doi: 10.7759/cureus.64722 39156376 PMC11328157

[17] Min S Zhou Y Sun Y Ye J Dong Y Wang X . Knowledge, attitude, and practice associated with antimicrobial resistance among medical students between 2017 and 2022: A survey in East China. Front Public Health. (2022) 10:1010582. doi: 10.3389/fpubh.2022.1010582 36353280 PMC9637849

[18] Bloom: Handbook I: Cognitive Domain - Google Scholar. n.d. Available online at: https://scholar.google.com/scholar_lookup?title=Handbook%20I:%20The%20Cognitive%20Domain&author=B.S.%20Bloom&publication_year=1956& (Accessed April 7, 2026).

[19] Efthymiou P Gkentzi D Dimitriou G. Knowledge, attitudes and perceptions of medical students on antimicrobial stewardship. Antibiotics. (2020) 9:821. doi: 10.3390/antibiotics9110821 33213047 PMC7698472

[20] Action Against Antimicrobial Resistance Requires a One Health approach. Available online at: https://iris.who.int/server/api/core/bitstreams/1f1c5d47-545b-407d-9784-e34b1b63d534/content (Accessed April 7, 2026).

[21] Dunn K Hamilton Hurwitz H Toledo JP Schwaber MJ Chu M Chou R . Summary of WHO infection prevention and control guideline for covid-19: striving for evidence based practice in infection prevention and control. BMJ (2024) 385:q645. doi: 10.1136/bmj.q645 38782413 PMC11116985

[22] Gupta MK Vohra C Raghav P. Assessment of knowledge, attitudes, and practices about antibiotic resistance among medical students in India. J Family Med Prim Care. (2019) 8:2864–9. doi: 10.4103/jfmpc.jfmpc_504_19 31681657 PMC6820394

[23] Antimicrobial Resistance Curriculum Assessment Tool for Medical Education. Available online at: https://iris.who.int/server/api/core/bitstreams/14ab727a-b6fe-4378-9be0-d287453be50e/content (Accessed April 7, 2026).

[24] Lubwama M Onyuka J Ayazika KT Ssetaba LJ Siboko J Daniel O . Knowledge, attitudes, and perceptions about antibiotic use and antimicrobial resistance among final year undergraduate medical and pharmacy students at three universities in East Africa. PLoS ONE. (2021) 16:e0251301. doi: 10.1371/journal.pone.0251301 33961678 PMC8104438

[25] Jairoun A Hassan N Ali A Jairoun O Shahwan M. Knowledge, attitude and practice of antibiotic use among university students: a cross sectional study in UAE. BMC Public Health. (2019) 19:518. doi: 10.1186/s12889-019-6878-y 31060543 PMC6501289

[26] Wang Y Guo F Wei J Zhang Y Liu Z Huang Y. Knowledge, attitudes and practices in relation to antimicrobial resistance amongst Chinese public health undergraduates. J Glob Antimicrob Resist. (2020) 23:9–15. doi: 10.1016/j.jgar.2020.07.023 32814152

[27] Sakr S Ghaddar A Hamam B Sheet I. Antibiotic use and resistance: an unprecedented assessment of university students' knowledge, attitude and practices (KAP) in Lebanon. BMC Public Health. (2020) 20:535. doi: 10.1186/s12889-020-08676-8 32306940 PMC7169022

