# Correction: Knowledge, attitudes, and practices regarding antimicrobial use and antimicrobial resistance among medical students and house surgeons in India: a call to reform medical curricula and training practices

**DOI:** 10.3389/fpubh.2026.1990902

**Published:** 2026-09-21

**Authors:** Shaheer Aboobacker, Ijyaa Singh, Gauri E. Yadav, Hyfa Mohammed Ali, Sudhi Nath, Sandhya S. Kulkarni, G. K. Mini, S. S. Lal

**Affiliations:** 1 ReAct Asia Pacific, Thiruvananthapuram, India; 2 International Health Policy Program Foundation, Nonthaburi, Thailand; 3 MIMER Medical College, Talegaon Dabhade, Pune, Maharashtra, India; 4 Global Institute of Public Health, Ananthapuri Hospitals and Research Institute, Thiruvananthapuram, India

**Keywords:** antibiotic use, antimicrobial resistance, antimicrobial stewardship, cross-sectional study, India, knowledge, attitude and practice, medical curriculum

Additional affiliation “International Health Policy Program Foundation, Nonthaburi, Thailand” was omitted for author Shaheer Aboobacker. This affiliation has now been added for author Shaheer Aboobacker.

The original article has been updated.

